# Enhanced Removal of Lead by Chemically and Biologically Treated Carbonaceous Materials

**DOI:** 10.1100/2012/604198

**Published:** 2012-05-02

**Authors:** Mohamed E. Mahmoud, Maher M. Osman, Somia B. Ahmed, Tarek M. Abdel-Fattah

**Affiliations:** ^1^Department of Chemistry, Faculty of Sciences, Alexandria University, P.O. Box 426, Alexandria 21321, Egypt; ^2^Department of Molecular Biology and Chemistry Christopher Newport University, Christopher Newport University, Newport News, VA 23606, USA

## Abstract

Hybrid sorbents and biosorbents were synthesized via chemical and biological treatment of active carbon by simple and direct redox reaction followed by surface loading of baker's yeast. Surface functionality and morphology of chemically and biologically modified sorbents and biosorbents were studied by Fourier Transform Infrared analysis and scanning electron microscope imaging. Hybrid carbonaceous sorbents and biosorbents were characterized by excellent efficiency and superiority toward lead(II) sorption compared to blank active carbon providing a maximum sorption capacity of lead(II) ion as 500 *μ*mol g^−1^. Sorption processes of lead(II) by these hybrid materials were investigated under the influence of several controlling parameters such as pH, contact time, mass of sorbent and biosorbent, lead(II) concentration, and foreign ions. Lead(II) sorption mechanisms were found to obey the Langmuir and BET isotherm models. The potential applications of chemically and biologically modified-active carbonaceous materials for removal and extraction of lead from real water matrices were also studied via a double-stage microcolumn technique. The results of this study were found to denote to superior recovery values of lead (95.0–99.0 ± 3.0–5.0%) by various carbonaceous-modified-bakers yeast biosorbents.

## 1. Introduction

Lead is one of the highly toxic heavy metals and widely characterized as the major source of water pollution. Lead is well recognized for direct and negative impact on the human health as well as biological organisms and ecological system [1, 2]. Lead poisoning in humans causes severe damage to the kidney, nervous system, reproductive system, liver, as well as brain and causes illness or death. Severe exposure to lead has been connected with sterility, abortion, stillbirths, and neonatal death [3–5]. The permissible level for lead in drinking water is 0.05 ppm according to the US Environmental Protection Agency (EPA). Therefore, the presence of very low concentration levels of lead in drinking water is considered as highly toxic and requires more efficient removal, extraction, and treatment methodologies [3].

 A number of well-documented methods are known and reported in the literatures for potential applications in removal, extraction, separation, and preconcentration of lead from various water matrices as well as other environmental samples. These include precipitation, ion exchange, coagulation, floatation, reverse osmosis, membrane filtration, and solvent extraction [4]. Among these methods, adsorption is highly effective and economical one [5]. Silica gels, activated alumina, metal oxides and hydroxides, zeolites, clay minerals, synthetic polymers, and carbonaceous materials, such as activated carbon and molecular carbon sieves, were used as efficient sorbents for water treatments [6, 7].

 The uses of carbonaceous sorbents for heavy metal removal from various matrices via adsorption were the subjects of a number of research papers. A comparative study of lead adsorption from aqueous solutions by raw and activated charcoals of *Melocanna baccifera* Roxburgh (bamboo) was reported [8]. Adsorption of lead from aqueous solutions onto a graphene layer (C*π* electrons) by using activated carbon and charcoal was investigated and reported [9]. Sorption potential of impregnated charcoal for removal of lead and other heavy metals from phosphoric acid was reported [10]. Activated carbon was prepared from *Enteromorpha prolifera* (EP) by reaction with zinc chloride and the physicochemical properties were characterized by thermal stability, zeta potential, and Boehm titration methods [11]. Kinetics, isotherms, pH, and ionic strength studies of Pb(II) sorption on activated carbon prepared from *Polygonum orientale* Linn were reported [12]. Batch sorption dynamics and equilibrium for removal of lead ions from aqueous phase using activated carbon developed from coffee residue activated with zinc chloride were also studied and evaluated [13]. A study was presented for the removal of lead(II) from wastewater by activated carbon developed from Tamarind wood by zinc chloride activation [14]. Studies on the removal of Pb(II) from wastewater by activated carbon developed from Tamarind wood activated with sulfuric acid was also reported [15]. Removal of copper(II) and lead(II) ions from aqueous solutions by adsorption on activated carbon from a new precursor hazelnut husks was developed [16]. Palm shell activated carbon was used as a good adsorbent for removal of lead from aqueous solutions [17]. Kinetics and equilibrium adsorption studies of lead(II) onto activated carbon prepared from coconut shell were reported [18]. A two-site adsorption model was used to describe the kinetics of adsorption and desorption of Pb(II) in aqueous solution on activated carbon [19]. Selective adsorption of lead on novel modified active carbons and marine algal biomass was presented [20]. Carbon nanotubes (CNTs) were used as adsorbent for study and evaluation of the adsorption thermodynamic, kinetic, and desorption studies of Pb^2+^ [21]. On the other hand, removal and extraction of lead and other heavy metals from different matrices were also accomplished by the use of various adsorbents [22–25].

 Hybrid biosorbents are recently reported by several research works and these types of combined solid materials are characterized by incorporation of various metal binding and chelating characteristics from multicombined species [26–28]. Hybrid carbon biosorbents can be prepared by surface enhancement of the carbonaceous materials such as activated carbon or modified activated carbon with other chelating functional groups in certain biological components for the formation of new hybrid biosorbents. Recently, baker's yeast, known as *Saccharomyces cerevisiae*, was surface immobilized on silica gel, Dowex anion exchanger and other solid supports are successfully used as efficient biosorbents for removal of heavy metal ions such as Cr(III), Cr(VI), Hg(II), As(III), and As(v) from various matrices [26–32].

 Removal of Pb(II) from industrial effluents by conventional treatment methods is commonly experienced with major problems and high difficulties in treating such wastewaters due to the matrix effect. Therefore, our purpose of this study is aimed at development of an effective and low-cost hybrid biosorbents for sorption, removal, extraction, and treatment of lead from water samples via immobilization of low-cost baker's yeast, as a source of chelating functional groups on oxidized-reduced activated carbon sorbents. The metal-biosorbent interaction processes and mechanisms were aimed to investigate and explore in this study via monitoring, evaluation, and optimization of the various experimental conditions as well as all controlling factors such as medium pH, reaction contact time, sorbent dose, initial lead ion concentration, and interfering ions on the sorption capability.

## 2. Experimental Section

### 2.1. Chemicals and Solutions

 Powder commercial active carbon was purchased from Adwic Chemicals, Egypt, and used as received. Baker's yeast was purchased from Starch and Yeast Co., Alexandria, Egypt. Nitric acid, acetic acid, sodium acetate trihydrate, lead(II) acetate trihydrate are all of analytical grade and purchased from Aldrich Chemical Company, USA, and other metal salts were purchased from BDH Limited, Poole, England.

 Buffer solutions (pH 1.0, 2.0, 3.0, 4.0, 5.0, 6.0, and 7.0) were prepared from 1.0 M hydrochloric acid solution and 1.0 M sodium acetate trihydrate solution by mixing the appropriate volumes of the two solutions and diluting to 1.0 L. The pH value of resulting solutions was adjusted by a pH meter. The metal ion solutions were prepared from doubly distilled water (DDW).

### 2.2. Instrumentation

 A Shimadzu Fourier Transform infrared spectrophotometer (FT-IR system-BX 0.8009) was used in the range 200–4000 cm^−1^ to acquire the FT-IR spectra of activated carbon, active carbon-modified-baker's yeast, oxidized active carbon, oxidized active carbon-modified-baker's yeast, reduced active carbon, and reduced active carbon-modified-Baker's yeast adsorbents. These sorbents were also imaged by the use of scanning electron microscope (JSM-5300, JEOL Ltd.). An ion sputtering coating device (JEOL-JFC-1100E) was used to coat the SEM specimens with gold to increase the conductivity. The pH measurements of buffer and metal ion solutions were carried out by using an Orion 420 pH-meter calibrated against standard buffer solutions of pH 4.0 and 9.2. Pb(II) concentration in various samples was determined by Shimadzu model AA-6650 atomic absorption spectrophotometer at the specified wavelength.

### 2.3. Synthesis of Hybrid Oxidized-Reduced Active Carbon-Modified-Baker's Yeast Biosorbents

 A sample of 75.0 g of commercial activated carbon was weighed and transferred to a 2.0 L beaker. 1.0 L of HNO_3_ (2 : 1 v/v ratio) used as an oxidizing agent was added and the reaction mixture was stirred for 4 h. Oxidized active carbon was filtrated, washed with distilled water, and dried at 100°C to produce sorbent (Ox-AC). 25.0 g of (Ox-AC) sorbent was weighed and mixed with 50.0 g of baker's yeast and the reaction mixture was mixed well in presence of 50.0 mL of DDW. The oxidized active carbon-modified-baker's yeast biosorbent (Ox-AC-BY) was left to dry in an oven at 60°C for 24 hrs. Reduced-active carbon (Rd-AC) sorbent was also prepared in a similar way. A sample of 75.0 g-active carbon was weighed and added to 1.0 L of Na_2_SO_3_ solution (1.0 Molar) and the reaction mixture was stirred for 4 h. Reduced active carbon was filtrated, washed with distilled, water and dried at 100°C to produce Rd-AC sorbent. 25.0 g of Rd-AC sorbent was weighed and mixed with 50.0 g of baker's yeast and the reaction mixture was mixed well in presence of 50 mL of DDW. The reduced active carbon-modified-Baker's yeast (Rd-AC-BY) was left to dry in an oven at 60°C for 24 hrs. The list and specifications of all hybrid chemically and biologically modified active carbon biosorbents are given in Table 1.

### 2.4. Lead Sorption in Various Controlling Experimental Factors

#### 2.4.1. Effect of pH

 Batch equilibrium technique was used to determine the metal sorption capacity values (*μ*mol g^−1^) of oxidized-reduced active carbon-modified-baker's yeast biosorbents [33]. In this method, 100 ± 1 mg of dry biosorbent was weighed and added to a mixture of 1.0 mL of 0.1 M Pb(II) and 9.0 mL of the selected buffer solution (pH 1.0–7.0) in a 50 mL measuring flask and this reaction mixture was shaken at room temperature for 30 min by an automatic shaker. After equilibration, the mixture was filtered and washed three times with 100 mL-DDW. The unbounded metal ion was subjected to complexometric titration using 0.01 M-EDTA solution or by atomic absorption spectrophotometric analysis.

#### 2.4.2. Effect of Contact Time

 Batch equilibrium technique was also used and applied to study the effect of shaking time intervals (1, 5, 10, 15, 20, 25, and 30 min) on the metal sorption capacity and extraction percentage values of lead according to the following procedure [34]. A sample of dry sorbent, 50 ± 1 mg, was added to a mixture of 1.0 mL of 0.1 M of Pb(II) and 9.0 mL of the selected buffer solutions (pH 2.0 and 7.0). This mixture was shaken for the selected period of time, filtered, washed with 100 mL DDW, and the unextarcted metal ion was determined.

#### 2.4.3. Effect of Biosorbent Dose

 The effect of biosorbent dose (25, 50, 100, 250, and 500 mg) was also studied by the batch equilibrium technique. A mixture of 1.0 mL of 0.1 M Pb(II) and 9.0 mL of buffer solution (pH 7.0) was added to the selected sorbent dose into a 50.0 mL measuring flask. These were then shaken at room temperature for 30 minutes by an automatic shaker. After equilibration, the mixture was filtered and washed three times with 100.0 mL-DDW.

#### 2.4.4. Effect of Initial Lead Concentration

 Batch equilibrium process was used to determine the effect of different concentrations of lead on the sorption capacity values. In this method, 100 ± 1 mg of the dry biosorbent was weighed and added to a mixture of 1.0 mL of different metal ion concentrations (0.010, 0.025, 0.050, 0.100, 0.250, and 0.500 M) and 9.0 mL of the selected buffer solution (pH 7.0) in a 50.0 mL measuring flask. The reaction mixture was shaken at room temperature for 30 min by an automatic shaker. After equilibration, the mixture was filtered and washed three times with 100.0 mL DDW.

#### 2.4.5. Effect of Interfering Ions

 The same procedures were also used to evaluate the effect of foreign ions on metal sorption capacity values of Pb(II) by oxidized-reduced carbon-modified-Baker's yeast biosorbents. A sample of dry biosorbent, 100 ± 1 mg, was added to a mixture of 1.0 mL of 0.1 M of Pb(II) and 1.0 mL of 0.1 M of the selected foreign solution (KNO_3_, NaCl, CoCl_2_, Ni(OAc)_2_, and CuCl_2_) and 9.0 mL of the optimum buffer solution. This mixture was shaken for 30.0 min, filtered, washed with 100 mL DDW, and the unextarcted metal ion was determined.

### 2.5. Potential Applications of Hybrid Active Carbon Biosorbents for Removal of Pb(II) From Water Samples

Removal and extraction of lead species from various water samples were performed according to the following procedure [35]. Real water samples (Alexandria drinking tap water and agricultural waste samples) were collected and a 1.0 L of each sample was spiked with ~1-2 ppm of Pb(II). A multistage microcolumn system was used and applied to evaluate the efficiency of hybrid active carbon biosorbents for solid-phase extraction of Pb(II) from real water matrices via multistage microcolumn technique. Each column stage was packed with 200 ± 1 mg of the selected sorbent and water sample was passed with a flow rate of 10 mL min^−1^ under air pressure. Effluent solution was collected, acidified with nitric acid, and subjected to atomic absorption spectrophotometric analysis of the free unextracted Pb(II). Real water samples were also subjected for atomic absorption spectrophotometric analysis before running over the column.

## 3. Results and Discussion

### 3.1. Surface Characterization

**Figure 1:**
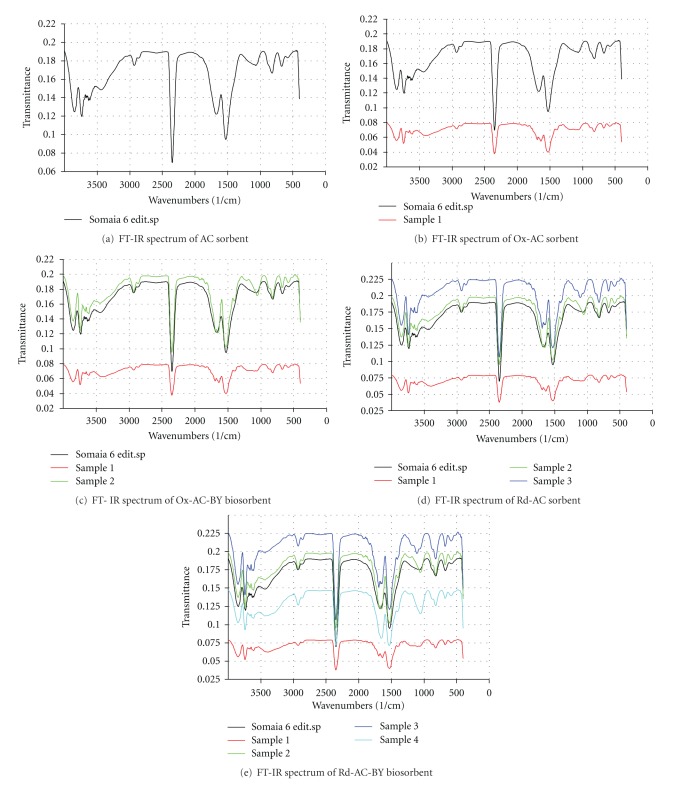
FT-IR spectra of various chemically and biologically treated carbonaceous sorbents.

 Surface functionality of carbon sorbents is characterized by its responsibility for all activity and reactivity as well as capability for all adsorption properties and processes. In this work, infrared spectroscopy was used to obtain information about the chemical structure and functional groups of the raw material and the hybrid activated carbon biosorbents. The FTIR spectrum represented in Figure 1(a) is showing few characteristic peaks that are mainly related to the organic nature of AC [36, 37]. However, upon redox treatments of AC, these peaks were intensified and other new IR peaks were produced. A peak centered at 1110–1120 cm^−1^ is mainly due to *ν* (C–O) of methoxy group. The strong bands appearing at 1520 and 1670 cm^−1^ are ascribed to the formation of oxygen functional groups based on highly conjugated C=O stretching in carboxylic or carboxylate group as well as carbonyl group. A band was observed at 2860 cm^−1^ and ascribed to the presence of aliphatic compounds. A broad band in the region 3300–3600 cm^−1^ is typically attributed to *ν* O–H stretching or adsorbed water molecule [37]. Thus, redox treatment process of AC-surface is important in improving and intensifying the various functional groups of AC sorbent. The FT-IR spectra of Ox-AC-BY and Rd-AC-BY biosorbents were also found to exhibit the same characteristic peaks of AC as well as some functional groups that are related to the yeast structure as previously reported [36].

**Figure 2:**
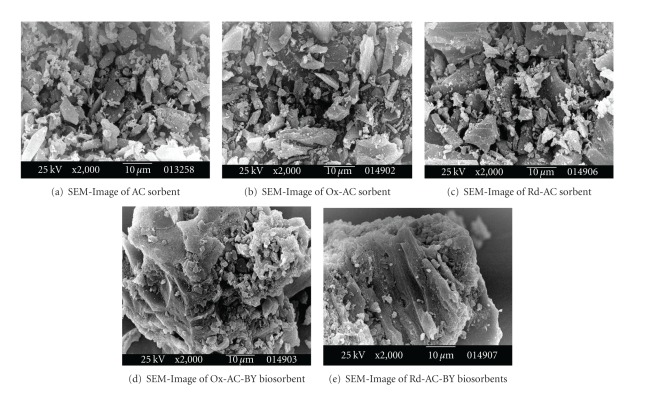
SEM-images of various hybrid chemically and biologically treated sorbents and biosorbents.

 Scanning electron microscopy (SEM) is a useful and efficient technique in evaluation of the surface morphology.  Figure 2(a) is the SEM-image taken for blank AC sorbent. Figures 2(b) and 2(c) are the SEM images of oxidized AC sorbent (Ox-AC) and reduced AC sorbents (Rd-AC), respectively. It is clear from these two images that the surfaces of both sorbents have been changed due to itching by oxidizing-reducing treatments. However, the particles have retained their original shapes and distribution. On the other hand, biological treatment of Ox-AC and Rd-AC with baker's yeast for the formation of Ox-AC-BY and Rd-AC-BY biosorbents, respectively, has resulted in pronounced surface changes of these two biosorbents. One can easily identify that biologically modified sorbents are existing in aggregate particles of Ox-AC and Rd-AC combined and coated with baker's yeast as evident from the SEM images and given in Figures 2(d) and 2(e).

### 3.2. Sorption Studies of Lead by Modified Active Carbon Sorbents

#### 3.2.1. Effect of pH

The effect of pH of tested metal ion solutions on the amount extracted by the modified-activated carbon sorbents and biosorbents is considered as one of the most important controlling factor in such procedure because of the liability of most metal ions to be strongly influenced by modified activated carbon biosorbents at certain pH values. Metal capacity (MC_*t*_) values at any time, expressed in *μ*mol/g, can be calculated from (2):

(1) $${\text{MC}}_{t}\;=\;\left[\frac{\left(C_{o}-C_{t}\right)V_{\text{mL}}}{{\text{Mass}}_{\text{g}}}\right]\times 1000,$$

where *C_o_* and *C_t_* are the initial and final metal ion concentrations in solution expressed in mol L^−1^·*V*_*mL*_ is the volume of metal ion solution expressed in mL, and Mass_g_ is the mass of sorbent or biosorbent expressed in gram. It is important to compare the identified metal sorption capacity values of modified active carbon sorbents with those determined for blank active carbon (AC).  Table 2 compiles the determined metal sorption capacity values, expressed in *μ*mol g^−1^ for Pb(II) under the effect of various buffer solutions (pH 1.0–7.0). It is evident from the listed data that AC is the lowest sorbent in interaction with lead under the effect of all tested buffer solutions. The maximum capacity value was found as 90 *μ*mol g^−1^ in buffer solutions with pH 5.0–6.0. However, treatment of active carbon by using nitric acid as an oxidizing agent for the formation of (Ox-AC) sorbent was found to increase the tendency and efficiency for lead removal in all studied acidic and neutral solutions. The maximum determined sorption capacity value was detected as 200 *μ*mol g^−1^ in neutral buffer solution with pH 7.0. In the same fashion, the metal sorption capacity of treated active carbon sorbent with sodium sulfite as a reducing agent for the formation of (Rd-AC) was also found to increase and reach a value of 280 *μ*mol g^−1^ in buffer solutions with pH 6.0–7.0.

 Further surface modification of AC, Ox-AC, and Rd-AC sorbents via loading of baker's yeast to produce the corresponding hybrid carbon biosorbents AC-BY, Ox-AC-BY, and Rd-AC-BY, respectively, were studied and compared for lead binding and sorption interaction processes. The determined metal capacity values are also listed in Table 2. It is evident from the given data that baker's yeast played a significant role in improving the sorption capacity values of all tested sorbents and biosorbents. A noticeable high increase in *μ*mol g^−1^ values is evident due to the presence of surface loaded yeast for AC-BY, Ox-AC-BY, and Rd-AC-BY in all examined buffer solutions. A gradual increase from 370 to 390 and 500 *μ*mol g^−1^ in the sorption capacity values of lead is evident by AC-BY, Ox-AC-BY, and Rd-AC-BY biosorbents, respectively, referring to the contribution of both hybrid chemical and biological treatments of AC. Among all studied and evaluated chemically and biologically treated active carbon sorbents, Ox-AC-BY stands as the highest biosorbent in lead sorption and uptake with a sorption capacity value of 500 *μ*mol g^−1^ in buffer solutions with pH 7.0.

#### 3.2.2. Effect of Shaking Time on the Metal Sorption Capacity

 The effect of shaking time is the second most important factor when batch or static technique is used in the processes of metal sorption capacity determination by newly synthesized sorbent or biosorbents. The results of this study are compiled in Table 3 and the values are expressed in *μ*mol g^−1^ as well as percent extraction. It is clear from the data listed in Table 3 that a 100.0% extraction of Pb(II) by Ox-AC and Rd-AC sorbents was obtained after only 20 minutes of shaking time in pH 2.0. On the other hand, immobilization of baker's yeast on the surface of both Ox-AC and Rd-AC sorbents for the formation of Ox-AC-BY and Rd-AC-BY biosorbents was found to slow down the sorption of lead. This trend is mainly due to the presence of excessive amount of surface active groups from both active carbon as well as yeast functional groups that are capable of sorption and interaction with lead. Therefore, a gradual increase in the percent extraction of lead by Ox-AC-BY and Rd-AC-BY biosorbents is evident in Table 3.

Ox-AC and Rd-AC sorbents were found to require 25 minutes to give a 100.0% extraction of lead. Ox-AC-BY and Rd-AC-BY biosorbents were characterized by their strong affinity for binding with lead in buffer solution (pH 7.0) and attaining their 100.0% extraction also at 30 minutes of shaking time. This trend indicates that higher pH values are the optimum solution for these four sorbents and biosorbents. Finally, the effect of shaking time was also compared with the results of active carbon-modified-baker's yeast AC-BY. AC-BY and were found to similarly behave toward lead sorption in both buffer solutions, pH 2.0 and 7.0. High percentage extraction values, 93.5 and 86.1%, were identified by this sorbent after 1.0 minute shaking time in pH 2.0 and 7.0, respectively. A 100.0% extraction of lead in both buffer solutions was obtained after 20.0 minutes of shaking time. Thus, one can conclude from the effect of contact reaction time that the presence of surface loaded baker's yeast on chemically treated active carbon and higher buffer solutions are all working together to favor fast equilibrium, interaction, and sorption of lead.

#### 3.2.3. Effect of Lead Concentration

 The study of sorption equilibrium isotherms is essential in supplying the required basic information for the design and operation of sorption equipments for wastewater treatment. Various isotherm models including Langmuir, Freundlich, and BET models have been investigated and evaluated to describe and predict the sorption isotherms. The objective of this section is to study the sorption of lead(II) ions from aqueous solutions onto modified activated carbon sorbents and biosorbents. Under these three models, an equilibrium condition must be established and a relationship exists between the concentration of the solution interacting species and the sorbed species. Langmuir adsorption model is based on assuming that the adsorptive forces are similar to the forces in chemical interaction. The Langmuir equation in the linearized form is given by (2):

(2) $$q_{e}=\frac{1}{q_{\max}}+\frac{1}{q_{\max}K_{\text{L}}}C_{e},$$

where *q_e_* (mg/g) is the equilibrium surface sorbed Pb(II), *C_e_* (mg/L) is the solution equilibrium concentration of Pb(II), *q*_max⁡_ (mg/g) is the maximum amount of Pb(II) which can be taken up by the sorbent, and *K*_L_ (L mg^−1^) is the Langmuir constant. On the other hand, the linearized Freundlich expression is given by (3):

(3) $$q_{e}=\log K_{\text{F}}+\frac{1}{n}\log C_{e},$$

where *K*_F_ and 1/*n *are the Freundlich constants. The Brauner-Emmet-Teller (BET) isotherm model proposes that the initial sorbed layer can act as a substrate for further sorption; then the isotherm, instead of leveling off to some saturated value at high concentrations, will be able to increase indefinitely. The simplified form of nonlinear form of BET is generally expressed by the following equation:

(4) $$q_{e}=\frac{q_{\max}K_{\text{B}}}{\left(C_{s}-C\right)\left[1+\left(K_{\text{B}}-1\right)\cdot C/C_{s}\;\right]\;}\cdot C_{e},$$

where *q*_*e*_ is the amount of sorbed Pb(II) per unit weight of activated carbon, *C*_*e*_ is the equilibrium concentration of Pb(II) in solution (mg/L or mol/L), *C*_*s*_ is the saturation concentration of the Pb(II), and *K*_B_ is a constant (function of energy of adsorption and temperature).

**Figure 3:**
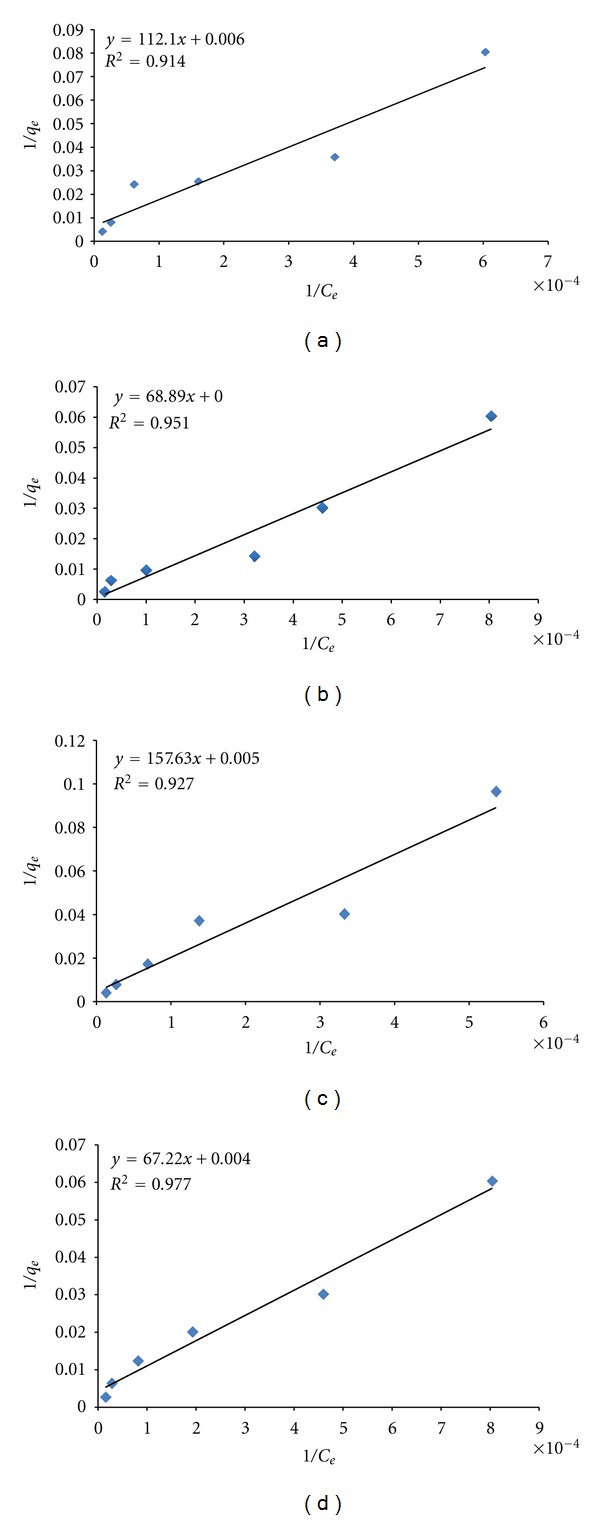
Langmuir's adsorption isotherms of lead sorption by hybrid active carbons (a) Ox-AC, (b) Ox-AC-BY, (c) Rd-AC and (d) Rd-AC-BY.

 Therefore, these three models have been used and applied to study and evaluate the sorption isotherms of lead by hybrid chemically and biologically modified active carbon.  Figure 3 shows Langmuir's adsorption isotherms of lead by the various hybrid active carbon sorbents and biosorbents. Straight lines were obtained in all cases indicating that sorption processes of lead by the studied sorbents and biosorbents were obeying Langmuir's adsorption isotherm model and the determined Langmuir's parameters are listed in Table 4. The high values of Langmuir's parameter, *q*_max⁡_, are clearly giving excellent evidences for the favorable sorption of lead by modified sorbents and biosorbents (Ox-AC, Rd-AC, Ox-AC-BY, and Rd-AC-BY).

**Figure 4:**
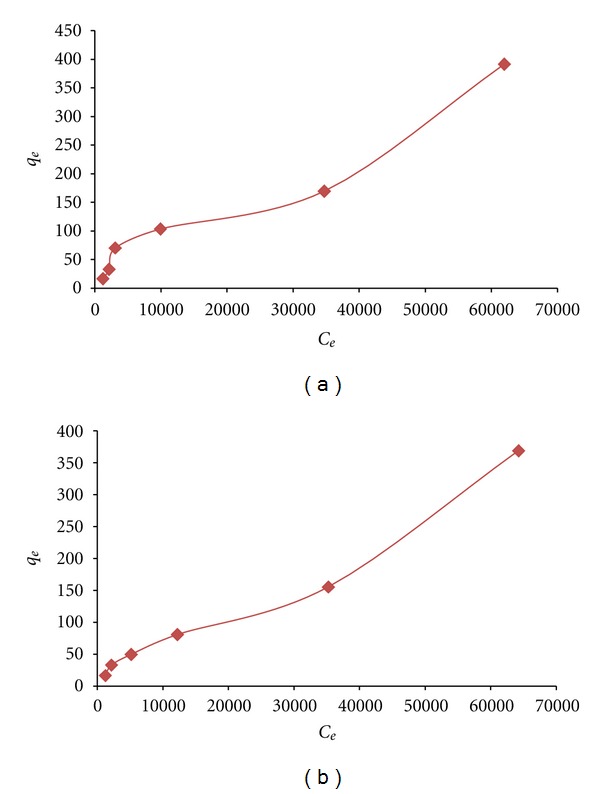
BET adsorption isotherms of lead sorption by hybrid active carbons (a) Ox-AC-BY and (b) Rd-AC-BY.

 In addition, the application of Brauner-Emmet-Teller (BET) isotherm model to the process of lead sorption by hybrid chemically and biologically modified active carbon sorbents and biosorbents was also studied. The collected results from applications of this model are represented by Figure 4 and clearly denote to the possible sorption of lead on the surface of these sorbents according to the postulates of BET adsorption models [38, 39].

#### 3.2.4. The Effect of Sorbent Dose

**Figure 5:**
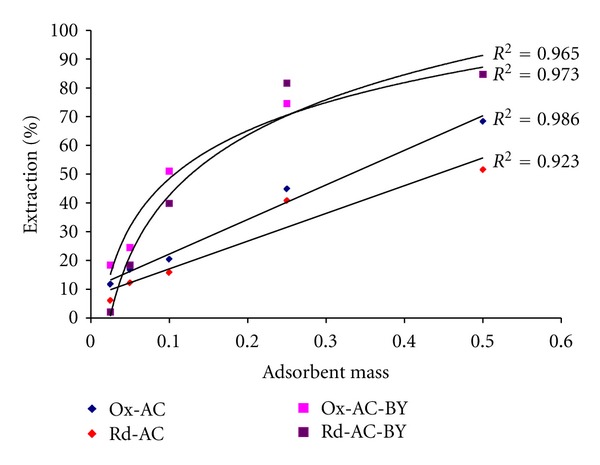
Effect of sorbent dose on Pb(II) sorption.

 The effect of sorbents and biosorbents dose was also studied as an important parameter to identify the relationship between lead sorption and mass of chemically and biologically modified sorbents.  Figure 5 represents such relationship between sorbent mass versus percent extraction of lead. It is evident from Figure 5 that the percentage extraction values of Pb(II) from aqueous solutions by all studied sorbents and biosorbents increase with the increase in the applied and selected mass. This trend is mainly due to the presence of more active surface sites and functional groups available to bind with Pb(II). The yeast-modified biosorbents, Ox-CY and Rd-CY, were found to exhibit their maximum percentage extraction values of Pb(II) (~100%) in presence of sorbent mass of ≥100 mg owing to the possible surface saturation of these sorbents with Pb(II). On the other hand, chemically modified Ox-AC and Rd-AC sorbents exhibited surface unsaturation trends upon their reactions with Pb(II) judging from the obtained straight lines [40]. 

#### 3.2.5. The Effect of Foreign Ions

 The interference of some foreign cations such as Co(II), Cu(II), Ni(II), and K(I) as well as anions (NO_3_^−^, Cl^−^, and acetate) on the process of lead extraction from aqueous solutions was studied and evaluated by all chemically and biologically treated active carbon sorbents and biosorbents. Evaluation of the possible interference of foreign ions was performed on the basis of equimolar concentration of lead acetate trihydrate versus other interfering species. The determined metal capacity values of lead in presence of these interfering anions and cations are listed in Table 5 along with those determined for lead capacity (Section 3.2). The existence of KNO_3_ and NaCl was characterized by low or no interfering effect in the sorption processes of lead by hybrid chemically and biologically modified active carbon sorbents and biosorbents. Ni(OAc)_2_ was found to exhibit low interfering effect owing to the determined metal capacity values of lead with respect to hybrid Ox-AC-BY and Rd-AC-BY biosorbents. CoCl_2_ and CuCl_2_ were found to show high interfering impact on the process of lead sorption by all modified active carbon sorbents. In the case of Ox-AC and Rd-AC sorbents, the capacity values of lead in presence of CoCl_2_ was found to decrease from 200 and 280 *μ*mol g^−1^ to 87 and 121 *μ*mol g^−1^, respectively, while in the case of CuCl_2_, these were found to reach 127 and 212 *μ*mol g^−1^, respectively. In addition, CoCl_2_ and CuCl_2_ were also characterized by high capability of direct interference in lead sorption processes by other modified AC sorbents, Ox-AC-BY and Rd-AC-BY, judging from the listed *μ*mol g^−1^ capacity values in Table 5. Thus, one can easily conclude that a strong competition between Pb(II), Co(II), and Cu(II) for binding with the active sites and functional groups loaded on the surface of chemically and biologically modified active carbon sorbents and biosorbents.

### 3.3. Applications of Hybrid AC Sorbents and Biosorbents for Extraction and Removal of Pb(II) from Water Samples

The capability of hybrid chemically and biologically modified active carbon sorbents and biosorbents to extract Pb(II) from real water samples (drinking tap water and Agricultural waste water**)** was further studied as the final step to explore the potential applications of these sorbents and biosorbents in water treatment and purification.  Table 6 compiles the results of metal removal and extraction via applications of a double-stage microcolumn technique. It is evident from the listed data that chemically modified active carbon sorbents are lower in their metal binding properties than other biologically modified biosorbents and both of them are higher than the blank AC sorbent. The application of AC sorbent as a packing material in a microcolumn extraction was found to produce a percentage extraction value of 70.0% after two runs of drinking tap water through the microcolumn. On the other hand, Ox-AC, Rd-AC, Ox-AC-BY, and Rd-AC-BY were found to produce percentage extraction values of 86.0, 80.5, 92.0, and 91.0%, respectively, for the first run. The second run was found to offer higher percentage extraction values of 91.8, 85.0, 99.0, and 95.7 for the same sorbents and biosorbents. The same trend and behavior were also identified when these modified sorbents and biosorbents were applied for lead removal from Agricultural waste water samples by producing percentage extraction values of the first run as 66.0, 62.0, 75.0, and 75.0 for Ox-AC, Rd-AC, Ox-AC-BY, and Rd-AC-BY, respectively. These values were found to increase when the second run was performed to produce 87.0, 83.0, 95.0, and 97.0%. Thus, it is important to point out here to the pronounced contribution of redox chemical treatments as well as surface immobilization of baker's yeast in the process of lead extraction and removal from the two examined water samples. The percentage extraction and removal values were found in the range of 95.7–99.0% for hybrid chemically and biologically modified Ox-AC-BY and Rd-AC-BY biosorbents. In addition, practical applications of double-stage microcolumn approach in lead(II) purification from water samples were also characterized and identified by providing excellent recovery results in the treatment process compared to single-stage microcolumn analysis.

## 4. Conclusion

 Several major findings and conclusions can be withdrawn from this study and these can be summarized in the following points. First, chemical treatments of commercial active carbon via simple oxidation and reduction reactions were found to improve the sorption efficiency and uptake of the chemically modified AC sorbents for lead. Second, oxidized active carbon-modified-baker's yeast biosorbent was characterized by its excellent sorption efficiency under the effect of all studied controlling factors compared to other chemically and biologically modified active carbon sorbents and biosorbents. Third, the equilibrium sorption of lead by chemically and biologically treated active carbon sorbents and biosorbents was found to follow typical adsorption isotherms and fitted well with both the Langmuir and BET adsorption isotherm models. Fourth, the determined lead sorption capacity values by chemically and biologically treated active carbon sorbents and biosorbents are higher compared to other previously reported methods. Fifth, excellent percentage recovery values of lead were obtained by the application of a double stage microcolumn separation technique.

## Figures and Tables

**Table 1:** Hybrid chemically and biologically modified-active carbon biosorbents.

Symbol	Sorbent
AC	Active carbon
Ox-AC	Oxidized active carbon by nitric acid
Rd-AC	Reducing active carbon by sodium sulfite
Ox-AC-BY	Oxidized active carbon by nitric acid and modified by baker's yeast
Rd-AC-BY	Reducing active carbon by sodium sulfite and modified by baker's yeast

**Table 2:** Sorption capacity values of lead by modified active carbon sorbents in various buffer solutions.

pH	Metal sorption capacity (*μ*mol g^−1^) in various buffer solutions*				
	AC	Ox-AC	Rd-AC	Ox-AC-BY	Rd-AC-BY
1	20	40	30	200	190
2	30	90	100	256	225
3	50	100	135	345	230
4	60	105	170	400	245
5	90	125	190	405	300
6	90	135	280	440	340
7	80	200	280	500	390

*Values are based on *n* = 3 with standard deviation of 2.0–4.0%.

**Table 3:** Effect of contact time on sorption capacity and percent extraction of lead by hybrid sorbents and biosorbents in pH 2.0 and 7.0 solutions.

pH	Min	Metal capacity in *μ*mol g^−1^ (percent extraction)*				
		Ox-AC	Ox-AC-BY	Rd-AC	Rd-AC-BY	AC-BY
2.0	1	20 (21.0%)	210 (79.0%)	100 (64.5%)	190 (84.4%)	290 (93.5%)
	5	55 (58.0%)	215 (81.0%)	120 (77.4%)	190 (84.4%)	290 (93.5%)
	10	75 (79.0%)	255 (85.0%)	130 (84.0%)	205 (91.0%)	300 (96.8%)
	15	85 (89.0%)	230 (86.8%)	145 (93.5%)	210 (93.0%)	300 (96.8%)
	20	95 (100.0%)	255 (96.0%)	150 (96.7%)	225 (100.0%)	310 (100.0%)
	25	95 (100.0%)	265 (100%)	155 (100%)	225 (100%)	310 (100.0%)
	30	95 (100.0%)	265 (100.0%)	155 (100.0%)	225 (100.0%)	310 (100.0%)

7.0	1	150 (75.0%)	450 (85.0%)	245 (87.5%)	320 (82.0%)	310 (86.1%)
	5	160 (80%)	425 (87.0%)	250 (89.0%)	335 (86.0%)	320 (88.9%)
	10	175 (87.5%)	435 (88.0%)	255 (91.0%)	340 (87.0%)	340 (94.4%)
	15	190 (95.0%)	440 (86.8%)	255 (91.0%)	365 (93.6%)	350 (97.2%)
	20	200 (100.0%)	470 (94.0%)	265 (94.6%)	375 (96.0%)	360 (100.0%)
	25	200 (100.0%)	470 (94.0%)	280 (100.0%)	385 (98.7%)	360 (100.0%)
	30	200 (100.0%)	500 (100.0%)	280 (100.0%)	390 (100.0%)	360 (100.0%)

*Values are based on *n* = 3 with standard deviation of 2.0–5.0%.

**Table 4:** The calculated Langmuir's parameters.

Adsorbent	*q*_ max_	*K*_ L_
Ox-AC	167	5.35236*E *− 05
Rd-AC	10000	1.45138*E *− 06
Ox-AC-BY	200	3.17259*E *− 05
Rd-AC-BY	250	5.95061*E *− 05

**Table 5:** Effect of foreign ions on the metal capacity values of lead.

Adsorbent	pH	*μ*mol g^−1^ capacity of lead in presence of interfering species					
		KNO_3_	NaCl	Ni(AcO)_2_	CoCl_2_	CuCl_2_	Pb(AC)_2_
Ox-AC	7.0	195	235	290	87	127	200
Rd-AC	7.0	250	200	265	121	212	280
Ox-AC-BY	7.0	480	510	299	126	290	500
Rd-AC-BY	7.0	350	400	309	183	290	390

*Values are based on *n* = 3 with standard deviation of 2.0–5.0%.

**Table 6:** Removal of lead from real water samples by modified AC.

Water sample	Sorbent	Sorbent mass	Spiked (ppm)	% Extraction	
				1st stage	2nd stage
Drinking tap water	AC			29.0%	70.0%
	Ox-AC			86.0%	91.8%
	Rd-AC		1.900	80.5%	85.0%
	Ox-AC-BY			92.0%	99.0%
	Rd-AC-BY	200 mg		91.0%	95.7%
Agricultural waste water	Ox-AC			66.0%	87.0%
	Rd-AC		1.590	62.0%	83.0%
	Ox-AC-BY			75.0%	95.0%
	Rd-AC-BY			77.0%	97.0%

*% Extraction** v**alues are based on *n* = 3 with standard deviation of 3.0–5.0%.
